# Recognizing Histopathological Simulators of Melanoma to Avoid Misdiagnosis

**DOI:** 10.7759/cureus.26127

**Published:** 2022-06-20

**Authors:** Sara Waqar, Sheeba George, Wilford Jean-Baptiste, Amina Yusuf Ali, Bithaiah Inyang, Feeba Sam Koshy, Kitty George, Prakar Poudel, Roopa Chalasani, Mastiyage R Goonathilake, Lubna Mohammed

**Affiliations:** 1 Research, California Institute of Behavioral Neurosciences & Psychology, Fairfield, USA; 2 Neurology, California Institute of Behavioral Neurosciences & Psychology, Fairfield, USA; 3 Research, California Institute of Behavioral Neurosciences & Psychology , Fairfield , USA; 4 Internal Medicine, Chitwan Medical College of Medical Science, Chitwan, NPL; 5 Pediatrics/Internal Medicine, California Institute of Behavioral Neurosciences & Psychology, Fairfield, USA; 6 Internal Medicine, California Institute of Behavioral Neurosciences & Psychology, Fairfield, USA

**Keywords:** recurrent nevus, reed nevus, acral nevus, congenital nevus, cellular blue nevus, deep penetrating nevus, dysplastic nevus, spitz tumors, ­melanoma and nevi, melanocytic nevus

## Abstract

Melanocytic lesions have a wide morphological spectrum, ranging from benign nevi to malignant melanoma. In contrast to a diagnosis of a benign nevus, a diagnosis of melanoma could mean intensive treatment, lifetime monitoring, and a worse prognosis. Therefore, melanocytic tumors are notoriously challenging and associated with a high risk of litigation in surgical pathology. After describing the basic features of nevi and melanoma, this article describes the detailed clinical and histological features of those lesions that share many similar features with melanoma. The entities included are Spitz nevi and atypical Spitz tumors (AST), Reed nevus, dysplastic nevus, cellular blue nevus (CBN), deep penetrating nevus, combined nevus, recurrent nevus, irritated nevus, congenital pattern nevus, acral nevus, and nevi of special sites. Knowledge of these imitators can help pathologists distinguish between benign and malignant cases and avoid misdiagnosis.

## Introduction and background

Melanoma is a malignant tumor developed from melanocytes [1]. Melanocytes are derived from neural crest cells. They are typically located in the basal layer of the epithelium, interspersed amongst the basal keratinocytes [2]. The incidence of cutaneous melanoma has significantly increased over the past decades. In 2022, the estimated new cases were 99,780, and the estimated number of deaths was 7,650 in the United States [3]. It is one of the deadliest skin cancers, and early diagnosis and treatment are critical in preventing disease progression. In advanced stages, the median survival time is less than one year [4].

Light-skinned individuals have a much higher incidence of melanoma than dark-skinned individuals [5-7]. Melanoma can be triggered by multiple risk factors such as genetic susceptibility, exposure to the sun, and artificial UV radiation like indoor tanning [8,9]. On the other hand, melanocytic nevi can show unusual histological features that may mimic melanoma. They can be seen as asymmetrical lesions with nonuniform nests of melanocytes, cytological atypia, and some mitotic activity. Some examples are nevi of special sites, Spitz nevus, cellular blue nevus (CBN), and dysplastic nevus [2,10]. Despite these characteristics that suggest malignant melanoma, these lesions have a benign course and do not require treatment [10].

The gold standard for diagnosing melanoma is pathological examination [11]. Over the last three decades, there has been a continuous change for improvement seen in diagnosing melanoma to diagnose it at an early stage. The ABCD mnemonic for dermoscopy, as well as computer-based technologies and genetic markers, have all contributed to the timely diagnosis of melanoma [11]. Nevertheless, the improved diagnostic criteria have also led to this pseudo-epidemic of melanoma because the incidence is rising but the mortality is relatively steady. One reason for constant mortality may be a regression of melanoma, but another reason is possibly overdiagnosis [12]. The main factor for overdiagnosis is the wide morphological spectrum of melanocytic tumors [13]. No single histologic criterion can be used to differentiate melanoma from a nevus, and all the histological features should always be seen in context with the clinical picture [14]. This article has reviewed the histopathological simulators of melanoma, highlighting the features that can create potential diagnostic pitfalls and lead to misdiagnosis.

## Review

Methods

We have done a literature search to find the most relevant published knowledge about histologic simulators of melanoma. Databases used for the search were PubMed, Google Scholar, Science Direct, Semantic Scholar, and ResearchGate. The search strategy included articles published in the last ten years and available as free full text for PubMed search. MeSh strategy/terms used was ((Melanoma) AND (Nevi)) AND ((("Nevus, Pigmented/diagnosis"[Mesh] OR "Nevus, Pigmented/pathology"[Mesh])) AND ("Melanoma/diagnosis"[Mesh] OR "Melanoma/pathology"[Mesh])). Keywords used for searching other databases included but were not limited to melanoma, melanocytic nevi, Spitz nevus, Reed nevus, pigmented spindle cell nevus, cellular blue nevus, deep penetrating nevus, congenital nevus, and dysplastic nevus.

Discussion

In dermatopathology, melanocytic pathology is one of the most challenging areas, and consequently, litigation risk is highest in this domain. The pathologist can overdiagnose melanoma as nevi and underdiagnose nevi as melanoma. This wrong diagnosis happens for different reasons, but most importantly, due to a lack of experience, not paying attention to fine details, not looking for mitosis, or ignoring them completely. Sometimes, not taking a second opinion from an expert dermatopathologist for hard-to-diagnose cases is also a cause of misdiagnosis [15]. The study by Piepkorn et al. concluded that expert consultation might help diagnose and manage difficult melanocytic lesions [16]. If we are aware of something, we can diagnose it with ease. Similarly, for melanocytic lesions, an accurate diagnosis can be achieved if we are well informed about the entity and have seen it before [15].

Importance of Clinicopathological Correlation

A good clinicopathological correlation is also one of the keys to an accurate diagnosis in this area of surgical pathology [15]. The clinical request form has important clues. The patient's age is one of the important factors, as melanoma is very uncommon in patients younger than ten years, except for large congenital neonatal melanocytic nevi. The pathologist should also keep the lesion site in mind, as some areas can display atypical morphological features and resemble melanoma. These body areas include acral skin, genital skin, milk line, flexural areas, ears, scalp, back and shoulders of elderly patients, and umbilicus [17]. The dermatologist gives a clinical impression of the lesion, and it should also be kept in mind. Generally, a pathologist should exercise extra caution in diagnosing the lesion as melanoma when a clinician thinks it is a nevus. However, sometimes the clinician diagnoses the lesion as melanoma, but the lesion has benign histological features [17]. Lastly, the size of the lesion and whether the biopsy is partial or complete are also essential factors impacting the report of a lesion [17].

For the reader to better understand the entities that resemble melanoma, the basics of nevi and melanoma are discussed briefly, followed by details of entities that resemble melanoma.

Features of Nevi

Melanocytic nevi are a diagnostic group of benign melanocytic tumors. If the nevus is located in the epidermis, it is called a junctional nevus; if in the dermis, it is called an intradermal nevus; and if present in both locations, it is called a compound nevus. The nevus cells predominantly tend to form clusters or nests in junctional nevi [2]. Single melanocytes trickle down between the collagen bundles in intradermal and compound nevi as they go deeper into the dermis. These cells also get dispersed from one another while going downwards. During this conversion from large nests to smaller nests and finally, into single cells, the nevomelanocytes or nevus cells tend to get smaller. This phenomenon is known as "normal maturation" or zonation [2]. Based on their changes in shape, these cells are labeled as types A, B, and C. Type A cells are the large epithelioid cells arranged in nests at the junction and in the superficial dermis and have oval to round nuclei and abundant pale pink cytoplasm. Type B cells resemble lymphocytes as they appear like small round blue melanocytes, but lymphocytes are smaller and have more dense chromatin than type B cells. If present abundantly, they can look like an inflamed nevus. Finally, type C cells are spindle-shaped cells and resemble Schwann cells. This neural appearance can make some areas look like neurofibroma [2].

**Figure 1:**
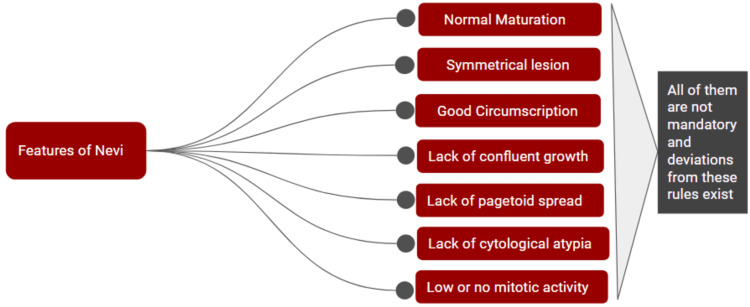
Histological features of nevi. This figure is created on Google Slides by Sara Waqar.

Benign lesions are symmetrical, which means the pattern of growth, cell density, and features are similar when one half of the lesion is compared to the other half. Junctional nests are of more or less the same size, and the pigment is equally distributed. Nevi are well-circumscribed, which means that the edges of the intradermal portion are well identifiable, most likely with similar-looking nests on both sides of the lesion [17]. There is a lack of confluent growth, which means that the junctional melanocytes are studded between the keratinocytes. If there are no intervening keratinocytes, then it indicates confluent growth. If melanocytes are present above the dermo-epidermal junction, it is called the "pagetoid spread." In nevi, melanocytes are commonly present in the basal layer of the epidermis, so there is usually a lack of pagetoid spread in nevi [2]. The cytology is observed at last, and usually, nevus cells have bland cytology. Mitosis can be seen in benign nevi but stays in the upper dermis. They are seen much more in younger patients than older, pregnant patients, and in lesions with a polypoidal growth pattern [17]. Not all of the above characteristics are mandatory, and there are exceptions to these rules [2]. Figure 1 describes the histological features of nevi.

**Figure 2:**
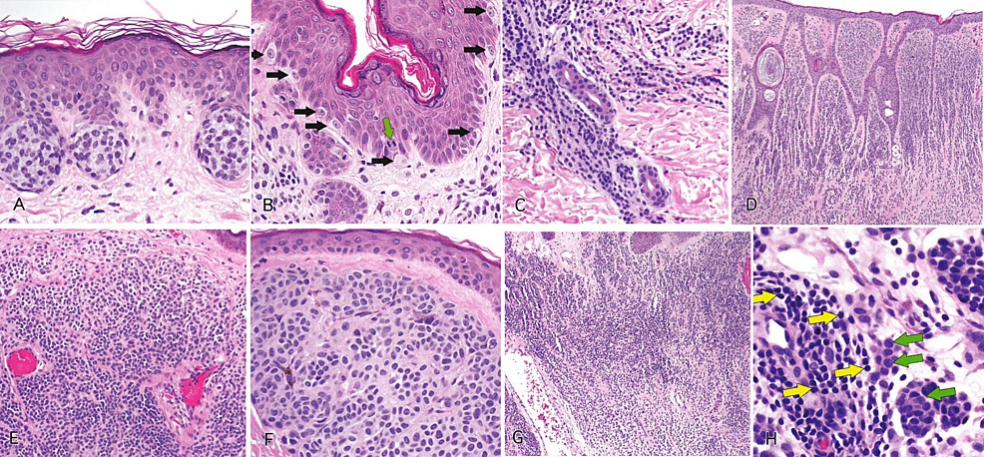
Slides showing histological features of nevi. Reprinted from Gardner J. Survival Guide To Dermatopathology, Pathology Survival Guides Series 1. Innovative Pathology Press, Arlington Virginia; 2019 [2] with permission from Copyright 2019 Innovative Pathology Press. (A) Nests of epithelioid melanocytes in a junctional nevus, arranged at the tips of rete ridges. (B) Melanocytes at the basal layer (black arrows) are spaced out, and there are intervening keratinocytes. Mitosis in a basal keratinocyte can be seen (green arrow). (C) Small clusters, cords, or single cells of dermal melanocytes can be seen trickling down the reticular dermis. (D) Shows normal maturation. Large nests in the superficial dermis transition to smaller ones, and finally, single cells are dispersed from one another in the deep dermis. (E) Type A melanocytes are large epithelioid cells at the top, and type B are small round blue cells at the bottom in the dermis. (F) Type A cells have oval to round nuclei with abundant pale pink cytoplasm, forming nests at the junction. (G) Type B melanocytes at low power, resembling lymphocytes. They can mimic an inflamed nevus. (H) At high power, type B melanocytes (green arrows) can be differentiated from lymphocytes (yellow arrows). Lymphocytes are smaller in size than type B melanocytes with darker chromatin.

Figure 2 demonstrates the histological features of nevi as seen in hematoxylin and eosin-stained slides [2].

Features of Melanoma

Melanomas are usually large lesions with a greater than 0.6 mm lateral diameter and depth going into the deep dermis. If a lesion is larger than 1 cm in its lateral diameter and its vertical dimension reaches the subcutis, in that case, it is probably a melanoma, with a few exceptions like congenital nevi, some blue nevi, Spitz nevi, and deep penetrating nevi. Melanoma also appears to have a disordered architecture with abnormal maturation, lack of symmetry, pagetoid spread, and confluent growth [18]. The cells are pleomorphic, which means the nuclear and cytoplasmic features of all the melanocytic cells are different from one another. Nuclei are enlarged, with coarsely clumped chromatin and prominent nucleoli. Some cells have abundant cytoplasm, and some have scanty amounts. Mitotic figures are present in the deep dermis, and if present along with cellular atypia and pleomorphism, it is a definite indication of melanoma [18].

**Figure 3:**
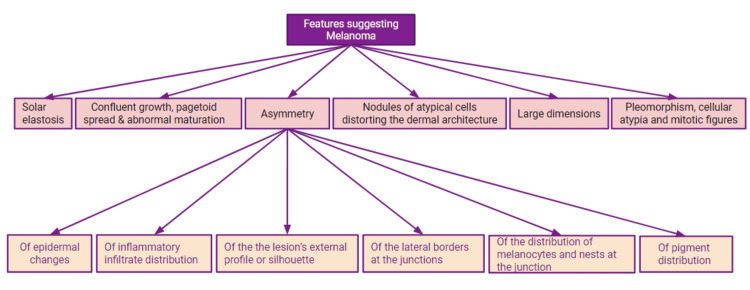
Histological features of melanoma. This figure is created on Google Slides by Sara Waqar [18].

It should be kept in mind that melanoma or nevi cannot be diagnosed solely based on the presence of one single feature. In other words, to diagnose melanoma, there is not a particular number of abnormal features or fixed criteria that are required for the diagnosis [2]. The pathologist must consider all the histological features and the clinical picture to make a final diagnosis. The rare variants should also be considered while diagnosing the lesion [2]. Figure 3 describes the important histological features of melanoma [18].

**Figure 4:**
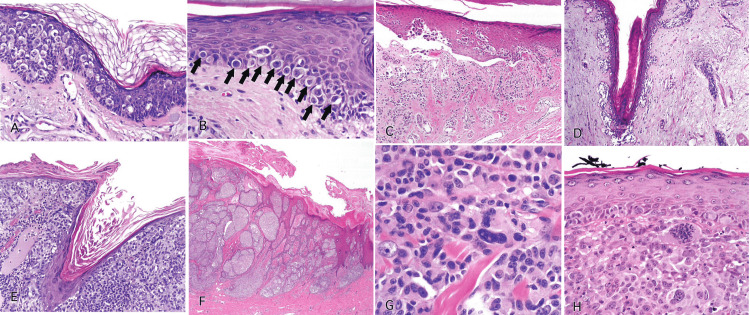
Slides showing histological features of melanoma. Reprinted from Gardner J. Survival Guide To Dermatopathology, Pathology Survival Guides Series 1. Innovative Pathology Press, Arlington Virginia; 2019 [2] with permission from Copyright 2019 Innovative Pathology Press. (A) Pagetoid spread is indicated by single melanocytes going above the basal layer of the epidermis into the upper spinous and even granular layer. (B) Confluent growth is indicated by single melanocytes crowding out the basal keratinocytes. (C) Acral lentiginous melanoma. The epidermis can be seen detaching from the dermis, giving the illusion of a blister. It is called an “unzipping” artifact, which indicates confluent growth. (D) Lentigo maligna type of melanoma. The melanocytes involve the hair follicle, trickling down along the basal layer into the follicle either as nests or single cells. (E) Phenomenon of “epidermal consumption.” Nests occupy the epidermis entirely, and there is only a thin strip of keratinocytes left in the epidermis above the melanocytes. (F) Abnormal maturation is present as large nests of melanocytes can be seen from top to bottom of the lesion. (G) Cytological atypia is present as cells are hyperchromatic and pleomorphic with prominent nucleoli. (H) Invasive melanoma. Sheets of pleomorphic cells with clumped chromatin and a prominent atypical mitotic figure in the dermis can be seen.

Figure 4 demonstrates the histological features of melanoma as seen in hematoxylin and eosin-stained slides [2]. 

The entities that resemble melanoma are described below.

Spitz Nevus and Atypical Spitz Tumors

In 1948, Sophie Spitz published her observations regarding melanomas in children. She pointed out that melanomas in children are rarely malignant compared to melanomas in adults [19]. These juvenile melanomas were subsequently considered benign and renamed Spitz nevi in honor of Dr. Spitz. It was also realized that Spitz nevi could also be found in adults [20].

Clinically, Spitz nevi are plaques, papules, or macules of different colors ranging from pink, red, tan, and brown, mainly affecting children and young adults and commonly present on the face and lower extremities. They are usually small (less than 0.6 cm) in size, solitary, and circumscribed lesions [20]. Histopathologically, Spitz nevi are composed of spindle and epithelioid cells. The epithelioid cells have abundant pale pink to gray cytoplasm. The nuclei are large, round, and have prominent nucleoli. These spitzoid features make them unique compared to other nevi. The cells can also have an elongated appearance with plump spindled nuclei. If these cells are vertically oriented, they give the impression of "bunches of bananas" or a "raining down" pattern [2]. Depending upon the location, they can be junctional, compound, or intradermal. In junctional Spitz nevi, nests of melanocytes are arranged at the junction. A clefting artifact can be seen separating these nests from the epidermis, which is usually acanthotic in Spitz nevi. The lesion is circumscribed, and nests are present at both edges rather than single cells, as seen in melanoma [2]. In the center of the lesion, the pagetoid spread of melanocytes, either as single cells or as nests, can be seen going into the epidermis. Another common finding is the presence of Kamino bodies, which are pink-colored basement membrane globules. Normal maturation occurs in the compound and intradermal Spitz nevi, which go deep and reach the reticular dermis [2]. A low mitotic rate (≤2/mm2) is seen, but even a high rate can be expected in children [20]. 

Spitzoid melanocytic lesions are a spectrum of lesions ranging from benign Spitz nevi to spitzoid melanoma [21]. Barnhill [22] divided these tumors into Spitz tumors without significant abnormalities, Spitz tumors with atypical features, and malignant melanoma. 

**Figure 5:**
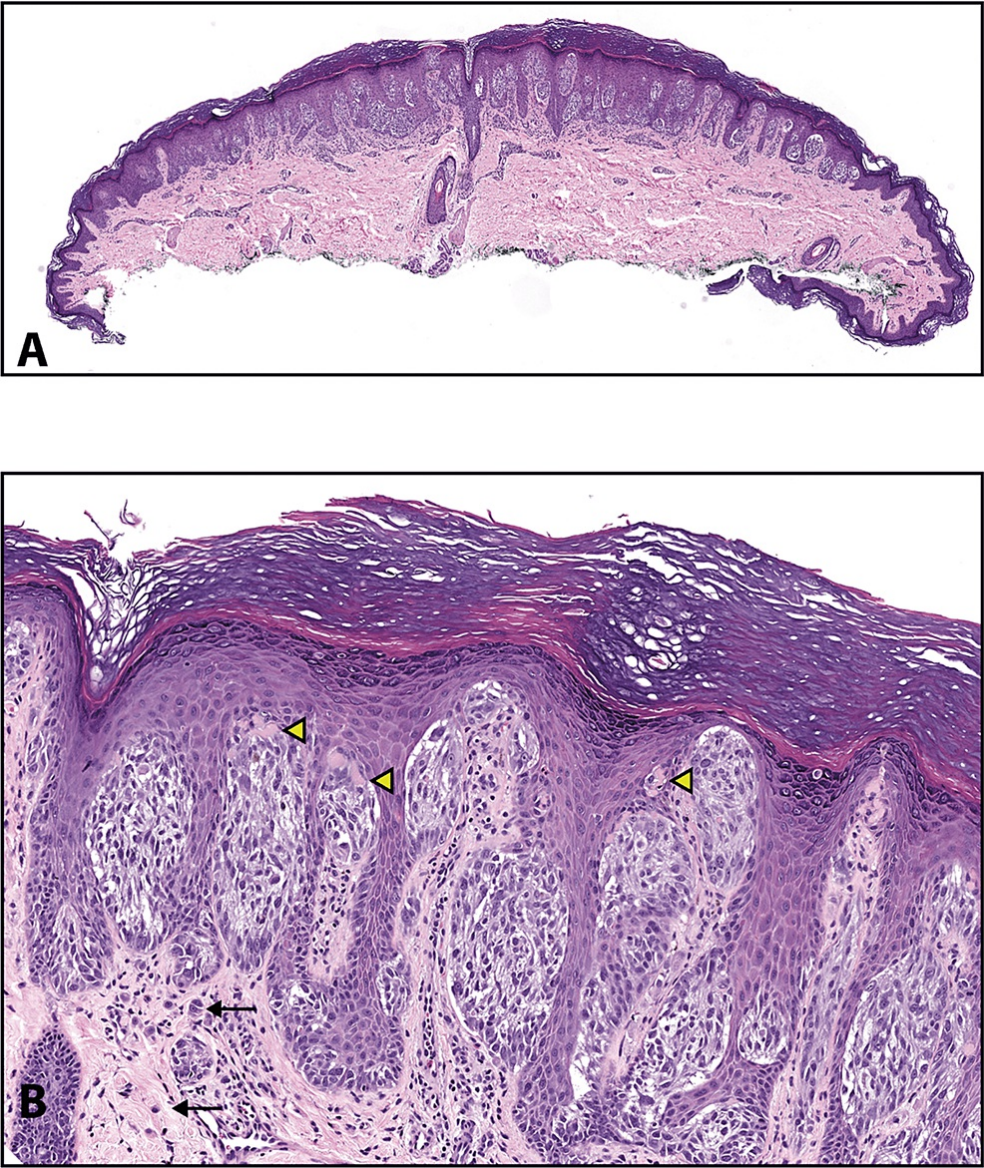
Spitz nevus. Reprinted from Harms KL, Lowe L, Fullen DR, Harms PW. Atypical Spitz Tumors: A Diagnostic Challenge. Arch Pathol Lab Med. 2015; 139(10): 1263-1270 [21] with permission from Archives of Pathology & Laboratory Medicine. Copyright 2010 College of American Pathologists. (A) This is the scanning magnification of a Spitz nevus. The lesion is symmetrical and predominantly nested with clefting artifacts around the nests. The epidermis is acanthotic (hematoxylin-eosin, original magnifications ×15). (B) This higher magnification shows large epithelioid melanocytes with prominent nucleoli. Kamino bodies are indicated with yellow arrowheads. Black arrows in the dermis point towards the nevomelanocytes that have retained their spitzoid morphology but have become smaller with their descent into the dermis (hematoxylin-eosin, original magnifications ×100).

Atypical Spitz tumors (AST) fall into the borderline category of this spectrum. They have spitzoid morphology but also other disturbing features like large size (≥10 mm), ulceration, significant depth, abnormal maturation, poor circumscription, cellular atypia, and an increased number of mitosis in the deeper parts of the lesion [2,17,21]. The biological potential of these tumors is very uncertain. In borderline cases, the sentinel lymph node can be positive, but the deposit is small, subcapsular, and resembles the lesion in its phenotype. However, if malignant features are present, like dense sheets of pleomorphic cells, it indicates a malignant potential [21]. It has now been found that different histological varieties of Spitz tumors are associated with specific genomic aberrations, and they can help differentiate benign from malignant neoplasms [23]. Figure 5 shows features of the Spitz nevus [21].

**Figure 6:**
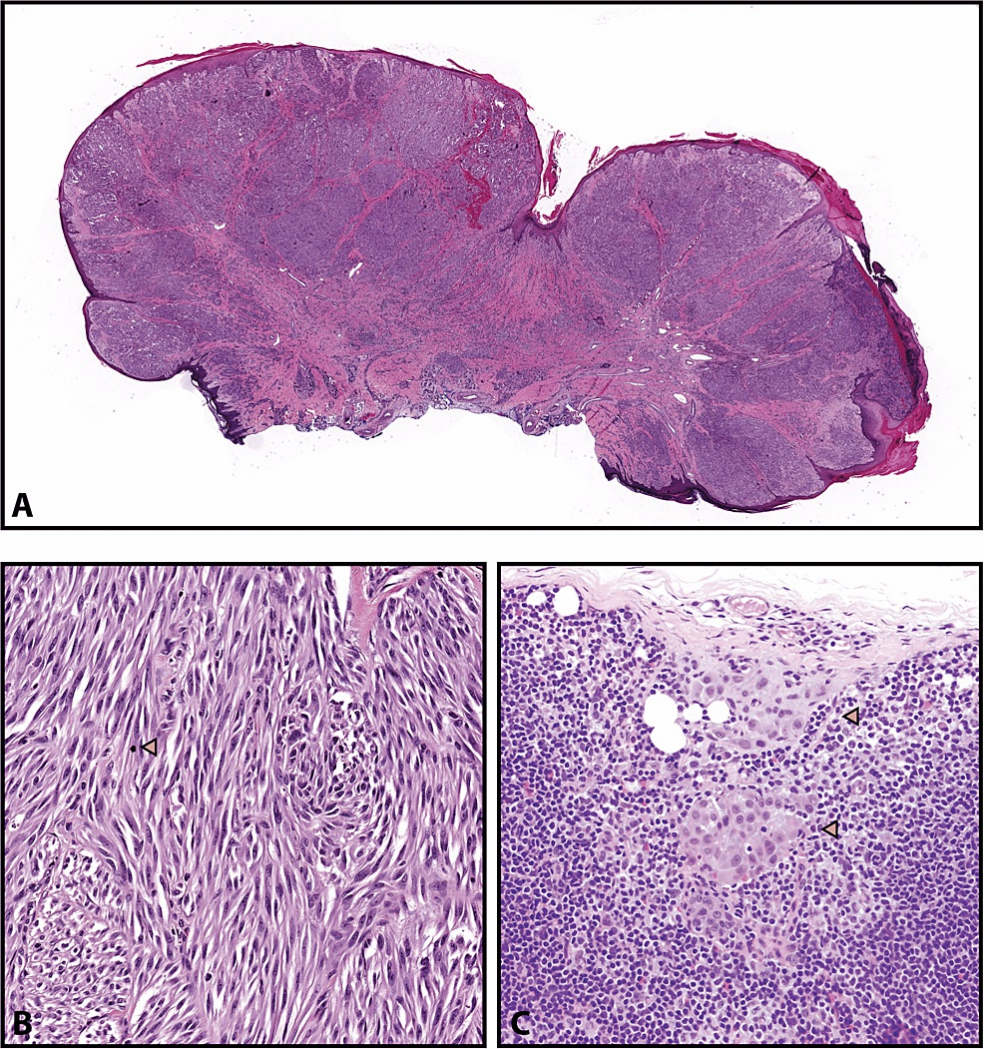
Atypical Spitz tumor. Reprinted from Harms KL, Lowe L, Fullen DR, Harms PW. Atypical Spitz Tumors: A Diagnostic Challenge. Arch Pathol Lab Med. 2015; 139(10): 1263-1270 [21] with permission from Archives of Pathology & Laboratory Medicine. Copyright 2010 College of American Pathologists. (A) This figure shows a large polypoid tumor with ulceration. (B) Spindled spitzoid cells are arranged in cellular fascicles with minimal pleomorphism and occasional mitotic figures (yellow arrowheads). (C) Yellow arrowheads show subcapsular deposits of spitzoid cells in a sentinel lymph node for an atypical Spitz tumor. These slides are stained with hematoxylin-eosin, seen in the above images at original magnifications ×5 (A), ×400 (B), and ×200 (C).

Figure 6 shows an atypical Spitz tumor at low and high magnifications [21].

Pigmented Spindle Cell Nevus (Reed Nevus) 

Reed nevus is considered a variant of Spitz nevus. They are small (≤7mm), dark brown to black colored plaques or papules with distinct borders [24]. Reed nevus is common in young adults and has female predominance, with the trunk and lower extremities being the most common sites [24]. Other rare sites are the conjunctiva and eyelids [25].

**Figure 7:**
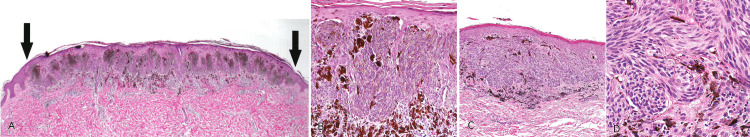
Reed nevus. Reprinted from Gardner J. Survival Guide To Dermatopathology, Pathology Survival Guides Series 1. Innovative Pathology Press, Arlington Virginia; 2019 [2] with permission from Copyright 2019 Innovative Pathology Press. (A) Reed nevus. A small, thin, and circumscribed lesion ends in a nest abruptly at both peripheral borders. (B) Junctional nests show vertically oriented spindle cells with fine melanin pigment. The nests of these cells are hanging down from the acanthotic epidermis like “bunches of bananas.” A melanophage band can be seen underlying the lesion, giving the lesion a dark pigmentation clinically. (C) Nests of spindle cells are sometimes seen running horizontally parallel to the epidermis from rete to rete, giving a “bridging” appearance similar to dysplastic nevus. (D) The melanocytes have plump spindled nuclei that resemble spindled melanocytes of Spitz nevus; that is why it is considered a variant of Spitz nevus. The underlying papillary dermis has darkly pigmented melanophages.

Histologically, there are overlapping features between Reed nevus and Spitz nevus. They are mostly found at the junction of the papillary dermis and are small, circumscribed, and thin lesions. The junctional melanocytes are plump spindle cells with abundant cytoplasm and fine melanin pigment. The nests can be oriented vertically, resembling "bunches of bananas," or run horizontally parallel to the epidermis, bridging the rete ridges like a dysplastic nevus [2]. The nests mostly end abruptly at both peripheral edges, except in growing pigmented spindle nevus, where single cells can be seen in the basal layer at the edges [18]. A central pagetoid spread of single melanocytes or nests of melanocytes can also be seen. Melanophages that are darkly pigmented can be seen in the papillary dermis. Cytological atypia can be seen but is not severe. The nuclei are hyperchromatic with occasional nucleoli, and mitotic figures are rare (<2/mm2). Differentiating this lesion from melanoma can be challenging because of its dark color, irregular margins, asymmetry, and cellular atypia [24]. Figure 7 depicts the histological features of Reed nevus [2].

Dysplastic Nevus

These nevi have a morphology and biology that makes them fall in the center of the spectrum of benign and malignant melanocytic tumors. Because of their unique nature, there has been much debate about various aspects of these lesions [26]. For example, controversy exists about their terminology. Some prefer the term "nevus with the architectural disorder" with a comment on melanocytic atypia, while others like "dysplastic nevus." [27]. Controversy exists regarding their biological potential as well. They are associated with an increased risk of melanoma, but most melanomas do not develop from dysplastic nevi. In this context, they can be considered non-obligate precursors of melanoma [26,28].

"Clark nevus" is another term used for dysplastic nevi [18]. It is because Clark et al. introduced the model of progression of nevus into melanoma and reported that melanoma-prone families have these unique moles, which he designated "The B-K mole Syndrome" [29]. Nowadays, dysplastic nevus syndrome, atypical mole syndrome, and familial atypical multiple mole melanoma syndrome (FAMMM) are terms used for patients with multiple clinically atypical nevi. These patients are at high risk for developing cutaneous melanoma [30].

Dysplastic nevi are large (>0.5 cm), flat lesions with irregular borders and variegated pigmentation. Histologically, they can be junctional or compound nevi. Compound nevi display "shouldering," which means that the junctional component is spreading to the peripheries beyond the dermal component, which is centrally located, thus forming "shoulders" [2]. The rete ridges are elongated, and the nests are connected at the tips of the rete, called "bridging." The bridging should not be confused with confluent growth, as the space between the rete at the top of the dermal papillae is not involved. Lamellar fibroplasia, which is layered pink collagen, can be seen in the papillary dermis wrapping around the rete ridges and nests of melanocytes that bridge between the rete. Sparse lymphocytic infiltrates and melanophages are found in the dermis [2]. Dysplastic nevi show cytological atypia-large melanocytes with brownish-grey cytoplasm, pleomorphic nuclei, and prominent nucleoli [31]. Assessment of atypia is very subjective and has been controversial, and several studies have been done regarding the grading of atypia in dysplastic nevi [24]. One observational study suggested that the risk of melanoma is higher with high-grade atypia [32].

**Figure 8:**
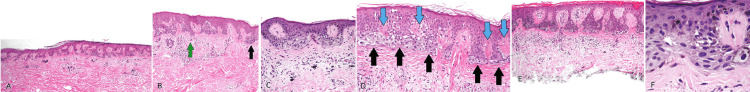
Dysplastic nevus. Reprinted from Gardner J. Survival Guide To Dermatopathology, Pathology Survival Guides Series 1. Innovative Pathology Press, Arlington Virginia; 2019 [2] with permission from Copyright 2019 Innovative Pathology Press. (A) A thin, broad lesion similar to its flat clinical appearance. The dermal component, if present, is in the papillary dermis and not in the deep dermis. (B) “Shouldering.” The green arrow points to the dermal component and the black to the junctional component. The junctional component extends to the periphery beyond the dermal component. (C) “Bridging.” The nests of melanocytes in the tips of rete ridges bridge across and are connected. Lamellar fibroplasia is seen underlying the nests and is wrapping around them. (D) Black arrows are pointing towards nests of melanocytes which are bridging across multiple adjacent retia and can be confused with confluent growth. The key here is that the inter-rete space above the papillary dermal tips (indicated by blue arrows) is uninvolved. (E) Lamellar fibroplasia. Bands of pink collagen wrap around the rete and nests of melanocytes that bridge between them. Lymphocytes can be seen scattered in the dermis. (F) Melanocytes are displaying cytological atypia. The nuclei of melanocytes are two to three times larger than the nuclei of spinous layer keratinocytes. The melanocytes have abundant grayish cytoplasm with fine dusty melanin pigment, giving it an appearance of “dirty dishwater.”

The distinction with melanoma is challenging based on all the above histological features, especially when the "dysplastic" features are developed sufficiently. Large size, architectural disorder, cellular atypia, and fusion of nests are all in common with melanoma [18]. Significant pagetoid single cells scattered at the border, diffuse cellular atypia, and dermal mitosis are all features indicating melanoma [17]. Figure 8 shows various features of the dysplastic nevus [2].

Cellular Blue Nevus

CBN is a variant of blue nevus and can have atypical features (atypical CBN), which can make their distinction from melanoma very challenging [33]. Blue nevi are immediately recognized because of brown melanin in the spindled melanocytes or intervening melanophages in the dermis. Reticular collagen bundles can be seen entrapped in the dermis in blue nevi [2].

Cellular blue nevi are small, dark blue nodules (0.5-1 cm) commonly seen in children or young adults [24]. They are intradermal nevi with one or more nodules extending deep into the dermis as bulging protrusions called "dumbbell pattern." They are more cellular in the deep aspect than the superficial part, and normal maturation cannot be seen [2]. The cellular part is formed by amelanotic spindle cells with large nuclei arranged in nests or fascicles. The conventional blue nevus part, composed of densely pigmented dendritic cells, surrounds the cellular part [2,24]. Background sclerosis can be seen along with possible degenerative changes [24]. Necrosis is generally considered absent in CBN and atypical CBN. Still, an unusual case of an atypical CBN with necrosis has recently been reported, simulating a "melanoma arising from blue nevus" [34].

**Figure 9:**
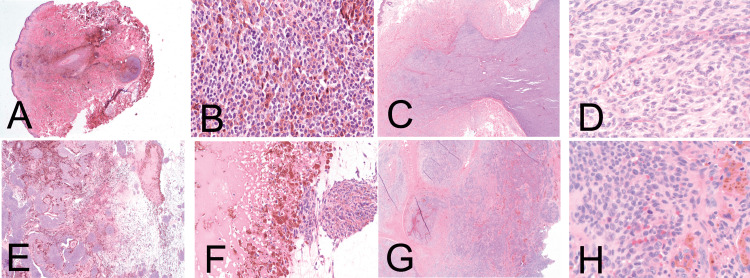
Cellular blue nevus. Reprinted from Zembowicz A, Phadke PA. Blue nevi and variants: an update. Arch Pathol Lab Med. 2011; 135(3): 327-336 [35] with permission from Archives of Pathology & Laboratory Medicine. Copyright 2010 College of American Pathologists. (A) & (B) CBN comprises small cellular nodules of epithelioid cells. (C) & (D) Dumbbell-shaped appearance of large CBN. At high power, it shows uniform oval cells with inconspicuous nucleoli and clear cytoplasm. (E) & (F) An area of cystic degeneration can be seen, which should not be confused with tumor necrosis. (G) & (H) Areas of hemorrhage and hemosiderin deposition in large atypical CBN. Hot spots of cellular atypia and increased mitotic activity can also be seen. These slides are stained with hematoxylin-eosin, seen in the above images at original magnifications ×20 (A, C, and E), ×400 (B, D, and H), ×200 (F), and ×100 (G).

Although CBN lacks overt atypia and high mitotic activity (usually <1/mm^2^ is present), atypical CBN shows cytological atypia but not to the malignant extent [35]. Melanoma can arise in the background of blue nevus or CBN and is recognized by marked atypia and mitotic activity (>3/mm^2^) [35]. Figure 9 illustrates the features of CBN [35].

Deep Penetrating Nevus

Plexiform spindle cell nevus, a term used by Barnhill et al., is another name for deep penetrating nevus [36]. This nevus extends deep into the dermis just like a cellular blue nevus, but instead of having a bulging "dumbbell" appearance in the dermis, it is a wedge-shaped lesion, like an upside-down triangle [2]. 

They are heavily pigmented black papules or nodules (< 1 cm) that typically affect younger patients (<40 years of age) [37]. Histologically, pigmented spindle cells with abundant cytoplasm and large nuclei are present in a deep penetrating nevus. The cells are arranged into fascicles and track the hair follicles, other adnexal structures, and neurovascular bundles, imparting a plexiform structure to the lesion [36].

**Figure 10:**
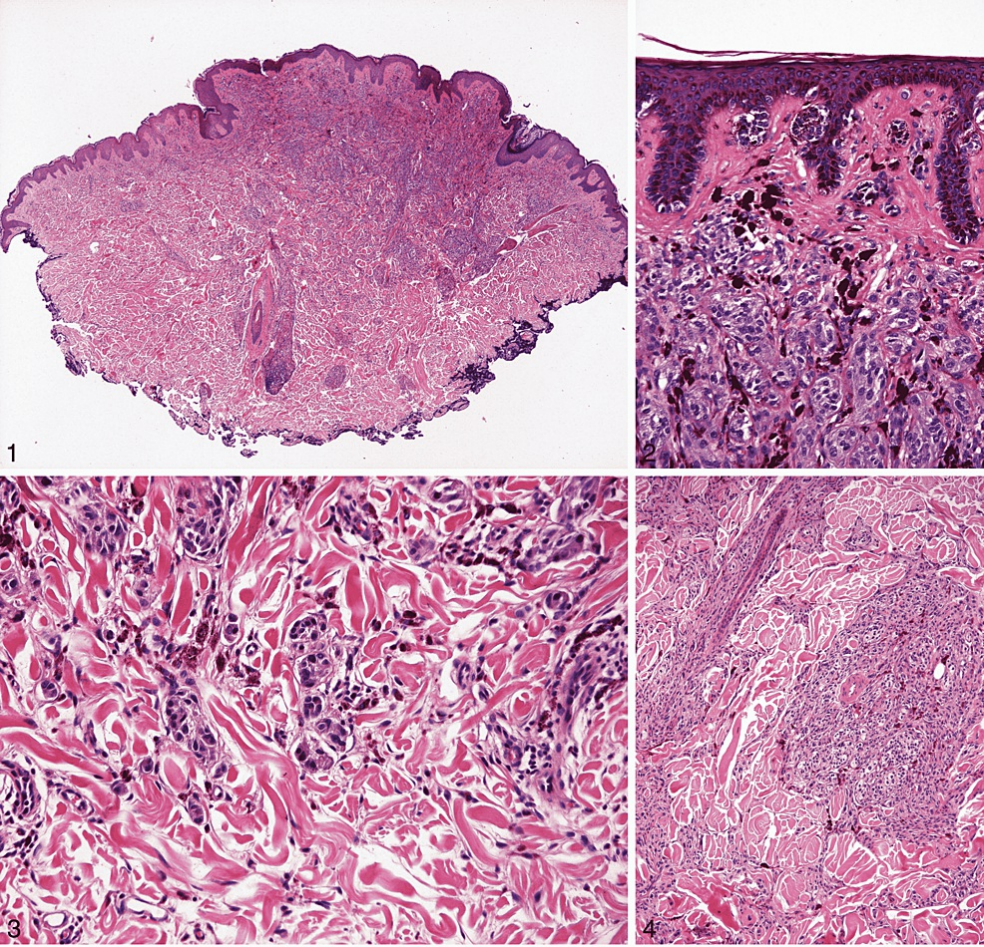
Deep penetrating nevus. Reprinted from Luzar B, Calonje E. Deep penetrating nevus: a review. Arch Pathol Lab Med. 2011; 135(3): 321-326 [37] with permission from Archives of Pathology & Laboratory Medicine. Copyright 2010 College of American Pathologists. (1) In this figure, the nevus shows a symmetrical wedge-shaped dermal proliferation. The lesion has a plexiform appearance due to several extensions going deep into the reticular dermis along the adnexa and neurovascular bundles (hematoxylin-eosin, original magnification ×40). (2) A junctional component is seen in most deep penetrating nevi, which shows nests and minimal lentiginous proliferation of melanocytes, just like in an ordinary nevus. There is a discontinuity between the junctional and the dermal component of the lesion in the papillary dermis (hematoxylin-eosin, original magnification ×200). (3) Discohesion of melanocytes is seen in the deeper aspects (hematoxylin-eosin, original magnification ×200). (4) Melanocytes grow along the skin adnexa and neurovascular bundles (hematoxylin-eosin, original magnification ×100).

Mild cytological atypia and pleomorphic nuclei can be seen along with rare mitosis in the deeper aspects. These features and a lack of maturation around the adnexa can become a pitfall and lead to the misdiagnosis of melanoma [37,38]. Figure 10 depicts histological features of a deep penetrating nevus [37].

Combined Nevus

Combined nevi are formed by combining two or more distinct types of nevus cells, giving the lesion an overall asymmetrical look, making it easy to misdiagnose a melanoma [39]. Different combinations can be seen, but the most prevalent is a combination of conventional nevus and blue nevus. A population of pigmented dendritic melanocytes can be seen mixed with conventional nevus cells. A particularly challenging combination is that of conventional and Spitz nevus because the presence of large epithelioid cells can lead to a diagnosis of melanoma [17]. Primarily, a component of conventional nevus is present, but other combinations also exist; for example, "BLITZ nevus," a combination of Spitz and blue nevus [17].

**Figure 11:**
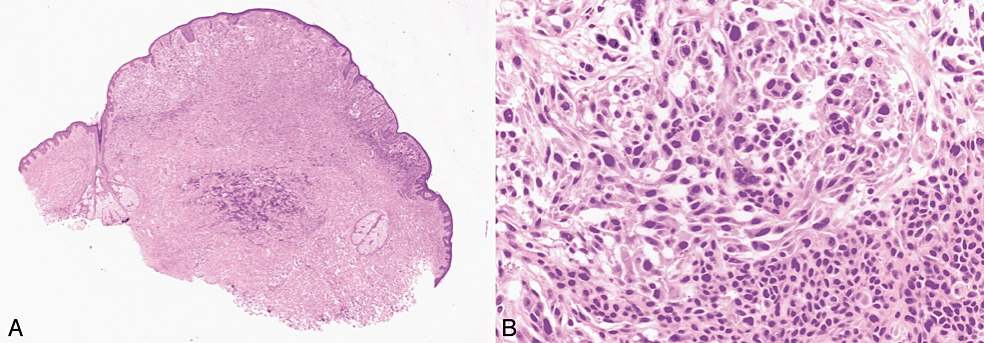
Combined nevus. Reprinted from Harvey NT, Wood BA. A Practical Approach to the Diagnosis of Melanocytic Lesions. Arch Pathol Lab Med. 2019; 143(7): 789-810 [17] with permission from Archives of Pathology & Laboratory Medicine. Copyright 2010 College of American Pathologists. (A) The lesion displays asymmetry, raising concern for melanoma developing within a nevus. (B) On high power, the deep penetrating nevus cells are in the upper panel, and the conventional nevus cells can be seen in the lower panel. Slides are stained with hematoxylin-eosin and can be seen at original magnifications ×20 (A) and ×400 (B).

There should be a concern about melanoma if there are findings like solar elastosis, pagetoid spread, pulverocyte-type cells, and melanoma in situ-like features in the epidermis. The lesion of a combined nevus may seem asymmetrical, but the symmetry becomes evident when considering each component individually [18]. A point to remember is that, in adult patients, it is very rare for a melanoma to develop in the dermal component of a nevus without an overlying melanoma in situ [39]. Figure 11 is an image of a combined nevus seen at low and high magnifications [17].

Recurrent Nevus

A recurrent nevus is a nevus that reappears or regrows after being previously excised. It looks like a new area of pigmentation within the previous site of the biopsy scar. This nevus frequently gets rebiopsied to rule out melanoma [2]. Recurrent melanocytic lesions may become challenging for a pathologist because they resemble melanoma [40] and also have been termed "pseudomelanoma" in the literature [41].

**Figure 12:**
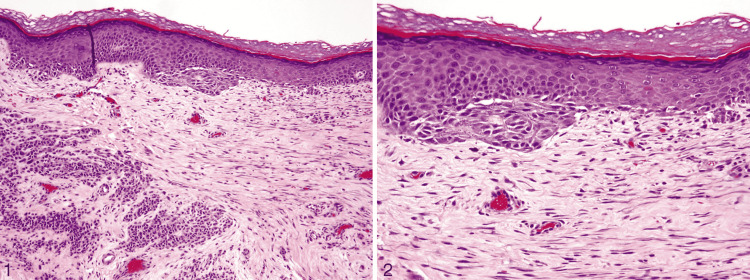
Recurrent nevus. Reprinted from Fox JC, Reed JA, Shea CR. The recurrent nevus phenomenon: a history of challenge, controversy, and discovery. Arch Pathol Lab Med. 2011; 135(7): 842-846 [41] with permission from Archives of Pathology & Laboratory Medicine. Copyright 2010 College of American Pathologists. (1) Trizonal pattern. The first one is atypically proliferating melanocytes and increased melanin at the dermo-epidermal junction; the second is fibrous tissue in the superficial and mid-dermis; the third is the congenital nevus at the base of the lesion (hematoxylin-eosin, original magnification ×100). (2) An architecturally disordered proliferation of melanocytes represented by confluent nests and single cells can be seen at the dermo-epidermal junction (hematoxylin-eosin, original magnification ×200).

Histologically, there can be junctional or compound melanocytic hyperplasia with effacement of the retiform epidermis; and junctional or compound melanocytic hyperplasia with retention of the retiform epidermis. All of these changes are associated with a dermal scar [42]. The melanocytes are arranged in nests or as single cells. A confluent growth pattern, pagetoid spread, and adnexal spread of melanocytes, with dermal inflammatory response and melanocytic atypia, can be seen. These histological features are also found in the intermediate and late stages of melanoma regression. In these cases, correlation with clinical findings and information about prior biopsy findings becomes very important [42]. Figure 12 depicts the features of recurrent nevus [41].

Traumatized or Irritated Nevus

The recurrent growth pattern described in recurrent nevus can also be seen in cases of nevi getting an excoriating injury by fingernails or getting a nick while using a shaving razor. Besides nevus cells, a superficial scar can also be seen [2]. There are single melanocytes scattered in the atrophic epidermis overlying the scar. Suppose there is just some scratching or rubbing but not complete epidermal ulceration; in that case, these nevi also show junctional features similar to recurrent nevus like pagetoid spread in the irritated zone [2].

Sometimes, it can become tough for even experts to differentiate a severely irritated nevus from melanoma [2]. A good clinical history of scratching or irritation can help in these cases. There are histologic clues such as parakeratosis, lichen simplex chronicus changes in the adjacent epidermis [2,17], focal fibrosis in the papillary dermis, pigment incontinence, and focal pagetoid spread in the central part of a conventional nevus [2].

Acral Nevus 

Nevi from acral skin, which includes palms, soles, and nail beds, can show unique histological features in their junctional component, which overlap with features of melanoma. Clinically, they are small (<6 mm) and well-circumscribed pigmented lesions [24].

**Figure 13:**
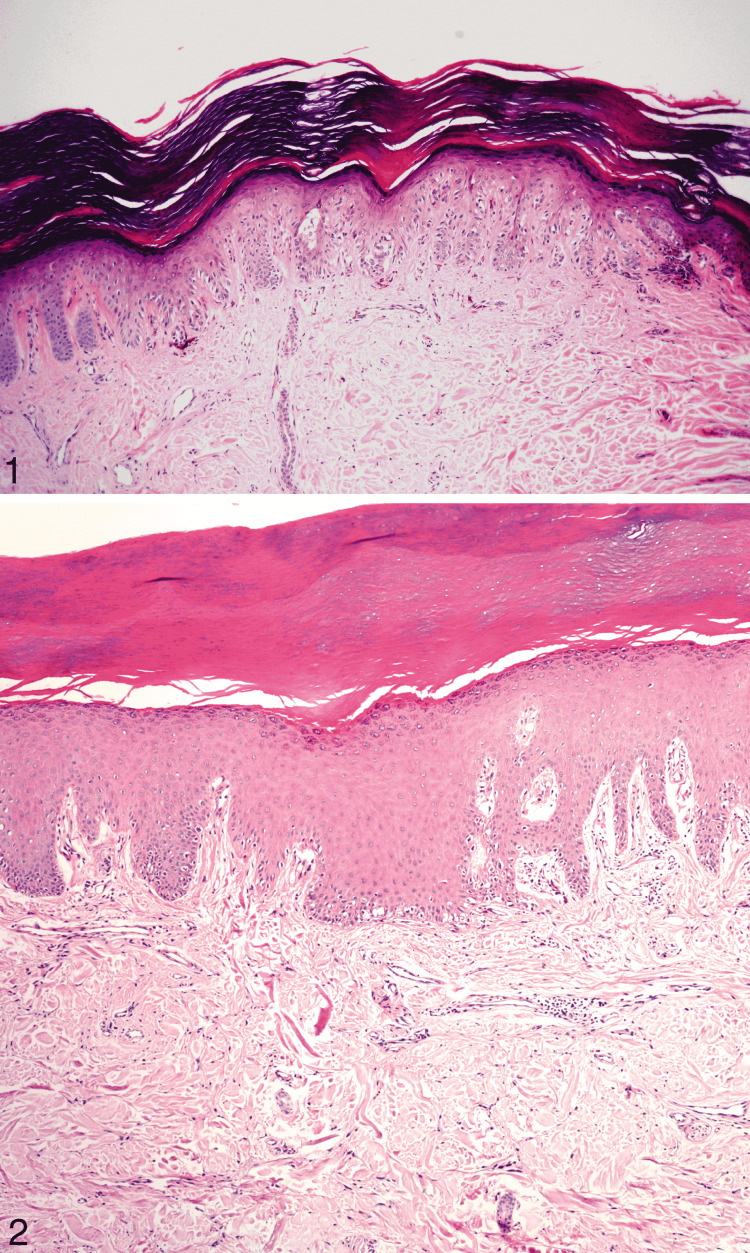
Acral junctional nevus and acral lentiginous melanoma. Reprinted from Bravo Puccio F, Chian C. Acral junctional nevus versus acral lentiginous melanoma in situ: a differential diagnosis that should be based on clinicopathologic correlation. Arch Pathol Lab Med. 2011; 135(7): 847-852 [44] with permission from Archives of Pathology & Laboratory Medicine. Copyright 2010 College of American Pathologists. (1) Medium power field of acral junctional nevus. Most cells are in solitary units, but they form groups at the base of the rete ridges (hematoxylin-eosin, original magnification ×100). (2) Acral lentiginous melanoma. Single melanocytes proliferate along the dermo-epidermal junction (hematoxylin-eosin, original magnification ×100).

These nevi can be compound or junctional [24]. There can be melanocytic nests or a lentiginous growth pattern with single melanocytes seen at the dermo-epidermal junction [2,24]. Cytologically, melanocytes appear to be round with prominent dendrites. They have larger nuclei than non-acral nevi and central punctate nucleoli. Pagetoid spread can be seen through the whole thickness of the epidermis [2,43]. Acral lentiginous melanoma can closely mimic acral nevus, especially near its periphery [2]. To avoid this pitfall, always consider the size of the lesion. If it is a large lesion and a partial biopsy was done, additional biopsies may be needed to rule out melanoma [2,44]. Figure 13 shows the acral junctional nevus (A) and acral lentiginous melanoma (B) [44].

Nevi of Special Sites

Nevi in specific areas in the body exhibit unique histopathological features which can be misinterpreted as malignant [45]. These areas include genitalia, ears, scalp, breast, flexural skin, shoulder, legs, and back [10]. These areas can demonstrate asymmetry, irregular nests, pagetoid spread, cytological atypia, and rarely, mitotic activity [2]. These features can raise a concern for melanoma, but these lesions are benign, and no intervention is required [10].

Nevi of the genitalia of young women can have a nodular appearance. If the patient is young, the lesion has a small size and symmetrical appearance; the junctional component is not extending laterally away from the dermal component; a normal maturation pattern is seen; then, most likely, the lesion is benign [17]. The junctional component is atypical, with variable sizes of nests growing together and forming large confluent structures [17]. There is also discohesion between cells of the atypical nests and clefting at the dermo-epidermal junction [46]. Melanocytes are the large epithelioid type with focal pagetoid spread [17]. As in all other nevi showing atypical features, if doubt is there, re-excision should be performed to ensure that the lesion is completely removed [17].

Congenital Pattern Nevi

The features of congenital nevi are unusual and can be confusing for an untrained eye, but these features are reassuring findings and indications of a benign lesion [2]. Congenital nevi have distinct histological features that are also present in noncongenital nevi. Therefore, the term congenital pattern is used, especially if the patient is an adult and it is not known whether the lesion was present at birth or not [2].

Melanocytes in congenital pattern nevi tend to grow into the deep dermis in a single filing pattern between reticular dermis collagen bundles. They are closely associated with dermal structures and track hair follicles, sweat glands, nerves, and blood vessels. Sometimes, they can be seen infiltrating the smooth muscle bundles of the arrector pili [2]. Melanocytes bulging into vessel lumens give an impression of "lymphovascular invasion." Still, it is not a real invasion, and therefore, there is no need to be alarmed. Large hyperchromatic cells can be seen in the dermis and mimic pleomorphism at low power. On high power, it becomes clear that they are indeed multinucleated nevus cells that have clusters of overlapping nuclei [2]. Cytological atypia, pagetoid spread, and architectural disorder are commonly seen in benign congenital nevi [17]. Exophytic papillomatous growth is sometimes seen as similar to that in seborrheic keratosis. In some nevi, adipocytes can be seen between the melanocytes in the dermis, and some nevi can be composed entirely of type C melanocytes, resembling neurofibroma [2]. In dark-skinned individuals, a band of darkly pigmented type A cells may be present [18].

**Figure 14:**
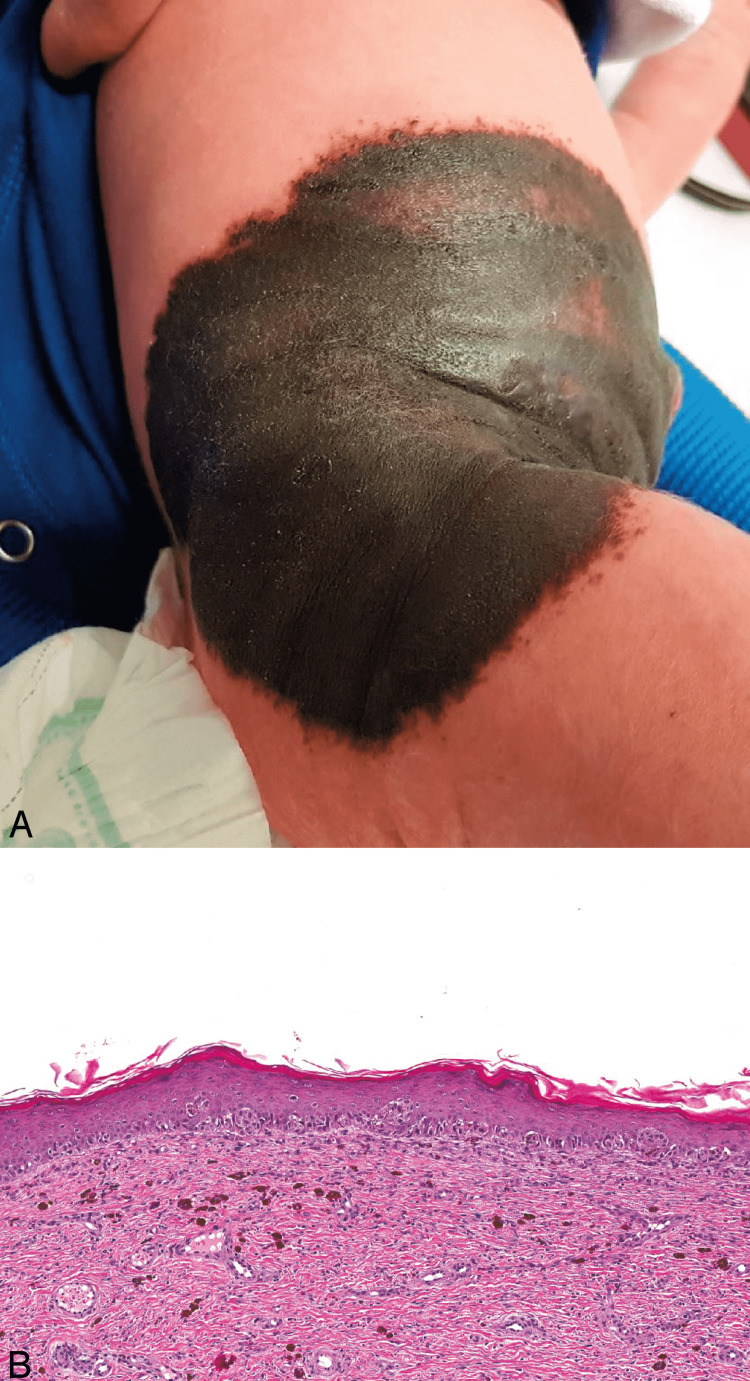
Giant congenital nevus. Reprinted from Harvey NT, Wood BA. A Practical Approach to the Diagnosis of Melanocytic Lesions. Arch Pathol Lab Med. 2019; 143(7): 789-810 [17] with permission from Archives of Pathology & Laboratory Medicine. Copyright 2010 College of American Pathologists. (A) A giant congenital nevus. (B) The junctional component shows atypia, which could be construed as melanoma in situ in an adult patient. In a neonatal setting, these changes are invariably benign (hematoxylin-eosin, original magnification ×84).

Giant congenital melanocytic nevi are greater than 20 cm in their largest dimension, and small nevi are less than 1.5 cm in the largest dimension [47]. The risk of malignant transformation in giant congenital nevi is associated with an increased risk of melanoma development in small ones is controversial [47]. Figure 14 illustrates a picture of a giant congenital nevus (A) and its histological features (B) [17].

Melanocytic Nevi in Pregnancy

If the lesion is from a woman of reproductive age and looks like an obvious nevus but has brisk mitotic activity, it is a question of whether the patient is pregnant [2]. Benign nevi in pregnancy can show characteristic features like increased dermal mitoses, a distinct histologic entity composed of round clusters of epithelioid melanocytes with prominent nucleoli, called superficial micronodules of pregnancy (SMOPs), and a higher Ki-67 proliferation index [48]. Mild junctional atypia, lentiginous growth patterns, confluent nests, and cellular discohesion are reported by some groups [17].

Table 1 summarizes the important features of the melanocytic lesions that resemble melanoma.

**Table 1:** This table summarizes the important histological features of melanocytic lesions that can simulate melanoma. Features of Spitz nevus, atypical tumors, pigmented spindle cell nevus, CBN, deep penetrating nevus, dysplastic nevus, recurrent nevus, nevi of special sites, and congenital nevi are summarized in this table for comparison [2,17,22,24,28,34-37,44].

Feature	Size	Lesional cells' shape	Nests	Confluent growth	Maturation pattern	Pagetoid spread	Cytological atypia	Other specific features
Spitz nevus [22]	Small, < 1 cm	Epithelioid and spindle-shaped - with abundant pale pink to gray cytoplasm, large oval nuclei, fine chromatin, and prominent nucleoli.	“Raining down” pattern and large nests with clefting artifacts.	Absent	Normal	In the center of the lesion	Absent	Kamino bodies
Atypical Spitz tumors [22]	Large, >10 mm	Spitzoid features	Have prominent cellularity	Present	Lack of maturation	It may be present over a large front.	Present	Ulceration; the biological potential is unknown; sentinel lymph nodes can be positive.
Pigmented spindle cell nevus [2]	Small, <7 mm	Pigmented, spindle shape	Either “bunches of bananas” or “bridging” pattern	Absent	Normal	Central	It may be seen but is not severe.	Darkly pigmented melanophages in the papillary dermis
Cellular blue nevus [24,34,35]	Small, 0.5 to 1 cm	Amelanotic oval to spindle cells	Intradermal with dumbbell pattern	They are intradermal lesions.	Lack of maturation	They are intradermal lesions.	Present in atypical CBN	Background sclerosis and degenerative changes
Deep penetrating nevus [2,36,37]	Small, <1 cm	Large pigmented spindle cells	Intradermal with wedge-shaped pattern	Absent	Lack of maturation	Absent	It may be present in deeper aspects.	Plexiform structure; nevus cells tend to track adnexal structures.
Dysplastic nevus [2,28]	Intermediate, >0.5 cm	Abundant grayish cytoplasm with fine dusty melanin	Bridging	Absent	Normal	Focal	Random, mild to moderate	Shouldering and lamellar fibroplasia. The biological potential is controversial.
Recurrent nevus [24]	Arises within the scar tissue	Junctional or compound melanocytic hyperplasia	Junctional or compound	Present	Atypical melanocytes are usually confined to the epidermis.	Focal	Present	Associated with a dermal scar and resembles melanoma with regression.
Acral nevus and nevi of special sites [17,44]	Small, <6 mm	Round with prominent dendrites in acral nevi, large epithelioid type in genital nevi	Nests or lentiginous growth	Present in genital nevi	Normal	Focal	Minimal	Acral nevus can resemble acral lentiginous melanoma.
Congenital nevus [2]	Small ones are <1.5 cm, and large ones are >20 cm.	Multinucleated nevus cells may be seen	Cells tend to grow in the deep dermis in a single filing pattern.	Absent	Normal	Present	The junctional component may show atypia.	Cells are closely associated with dermal structures. It may resemble melanoma in situ if junctional atypia is present.

Limitations

This review has to be seen in light of some limitations. It only describes the histopathological features of benign nevi that resemble melanoma. It does not include the different types of melanomas and their features, as some melanomas can look deceptively benign, creating a possibility of underdiagnosis. This review does not discuss borderline melanocytic lesions (for example, melanocytoma) with intermediate malignant potential. This review has not included results of ancillary studies like immunohistochemistry, comparative genomic hybridization, fluorescence in situ hybridization, and imaging spectrometry. These studies can be needed in cases where diagnosis alone on H&E sections becomes very hard.

## Conclusions

Benign melanocytic nevi and malignant melanoma share many histological characteristics, leading to confusion about the diagnosis. For example, atypical Spitz tumors can have abnormal maturation, poor circumscription, and cellular atypia; cellular blue nevus, deep penetrating nevus, and combined nevus can show unusual maturation patterns; dysplastic nevi display "bridging" of the rete ridges which can resemble confluent growth; nevi of special sites can demonstrate asymmetry and cytological atypia. The features of these entities are deceptively alarming, but these lesions are benign and do not need aggressive treatment. All the histological features should be seen in the clinical context and never alone. Familiarity with these lesions will avoid the misdiagnosis of melanoma.
